# Supplementary material for: Testing Modeling Assumptions in the West Africa Ebola Outbreak
Source: Sci Rep. 2016 Oct 10;6:34598. doi: 10.1038/srep34598 (PMC5056384; doi:10.1038/srep34598)
Supplement: Supplementary Information [file srep34598-s1.pdf]

Supplimentary Information

Testing Modeling Assumptions in the West Africa Ebola Outbreak

Authors: Keith Burghardt, Christopher Verzijl, Junming Huang, Matthew Ingram, Binyang Song, and Marie-Pierre Hasne

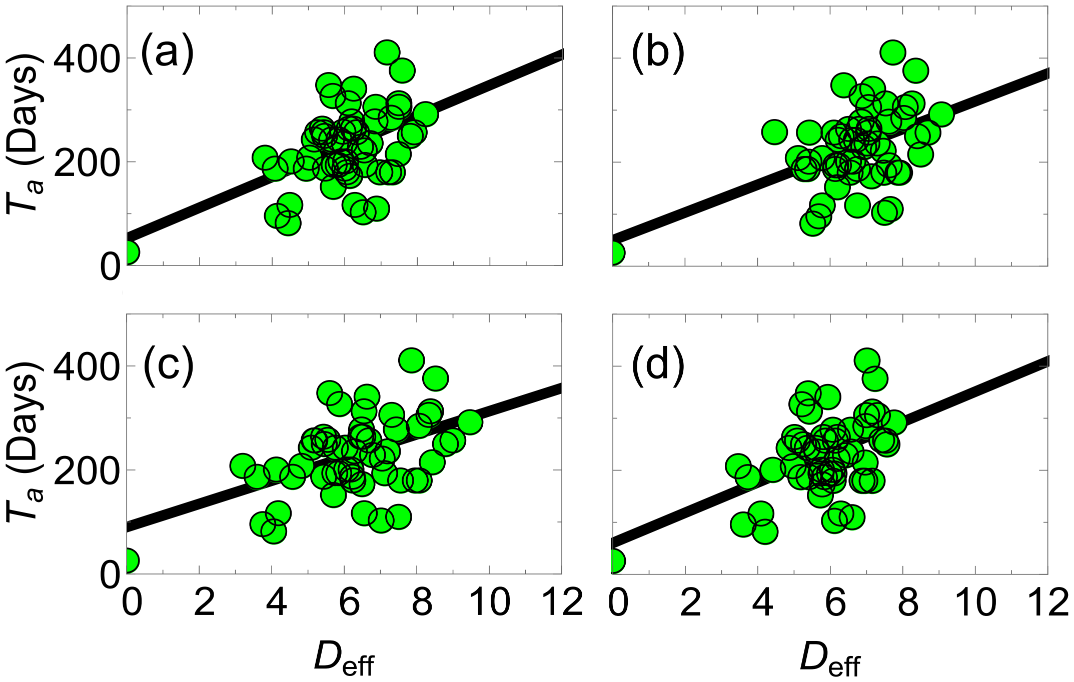

Figure S1. Arrival time (in days) versus the effective distance, using gravity migration models created in: Wesolowski, A. et al. Commentary: Containing the ebola outbreak – the potential and challenge of mobile network data. PLoS Currents Outbreaks (2014). Migration models with parameters based on (a) Cote d'Ivoire, (b) IPUMS, (c) Kenya, and (d) Senegal datasets. Correlations are all statistically significant, with $p < 10^{-4}$.

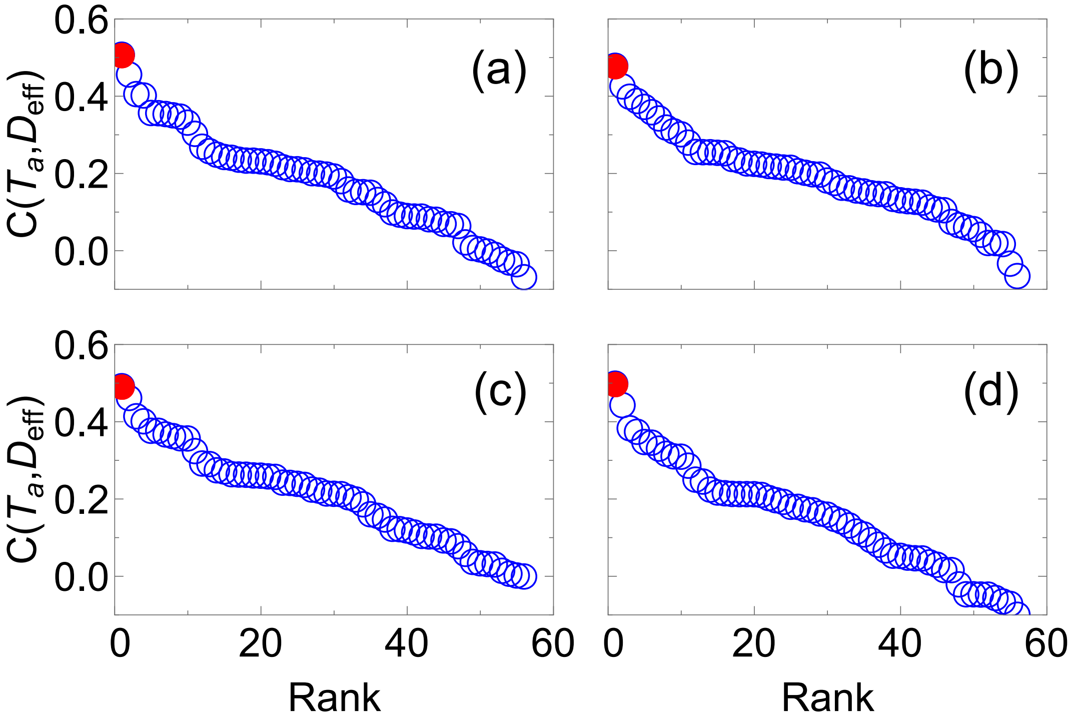

Figure S2. The correlation between arrival time (in days) versus the effective distance, using migration models created in the

previously cited paper, with various administrative divisions as candidate infection sources. In red is the true source of the infection, Guéckédou, Guinea, which consistently has the highest correlation. Migration models shown here have parameters based on (a) Cote d'Ivoire, (b) IPUMS, (c) Kenya, and (d) Senegal datasets. Using the bootstrapping method described in our paper, we find the correct origin 68% $\pm$ 1.5%, 63% $\pm$ 1.5%, 44% $\pm$ 1.6%, and 70% $\pm$ 1.4% of the time, respectively ($n = 56$, where we bootstrap 10^3 times).

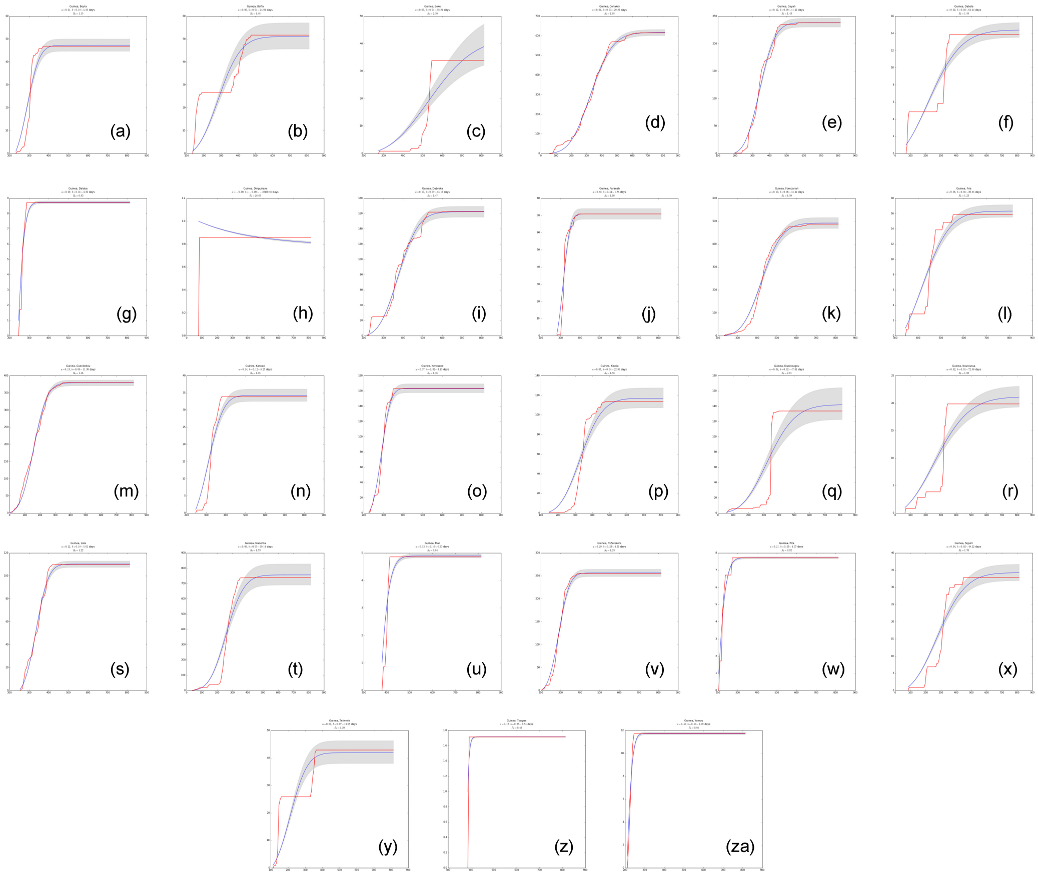

Figure S3. The best fit for the SDIR model for all infected prefectures of Guinea. In order, these are: (a) Beyla, (b) Boffa, (c) Boké, (d) Conakry, (e) Coyah, (f) Dabola, (g) Dalaba, (h) Dinguiraye, (i) Dubréka, (j) Faranah, (k) Forécariah, (l) Fria, (m) Guéckédou, (n) Kankan, (o) Kérouané, (p) Kindia, (q) Kissidougou, (r) Kouroussa, (s) Lola, (t) Macenta, (u) Mali, (v) Nzérékoré, (w) Pita, (x) Siguiri, (y) Télimélé, (z) Tougué, and (za) Yomou.

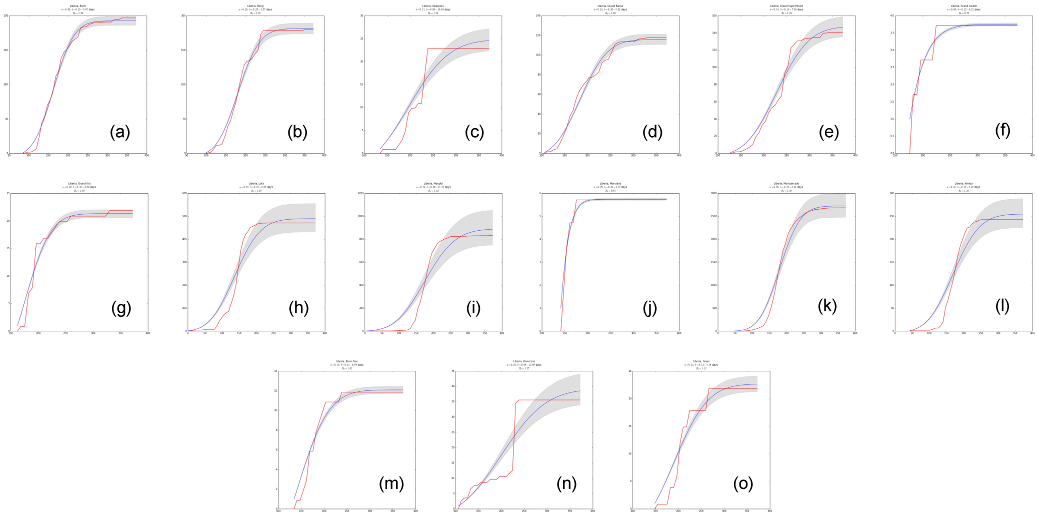

Figure S4. The best fit for the SDIR model for all first administrative level divisions in Liberia. In order, these are: (a) Bomi, (b) Bong, (c) Gbarpolu, (d) Grand Bassa, (e) Grand Cape Mount, (f) Grand Gedeh, (g) Grand Kru, (h) Lofa, (i) Margibi, (j) Maryland, (k) Montserrado, (l) Nimba, (m) Rivercess, (n) River Gee, and (o) Sinoe.

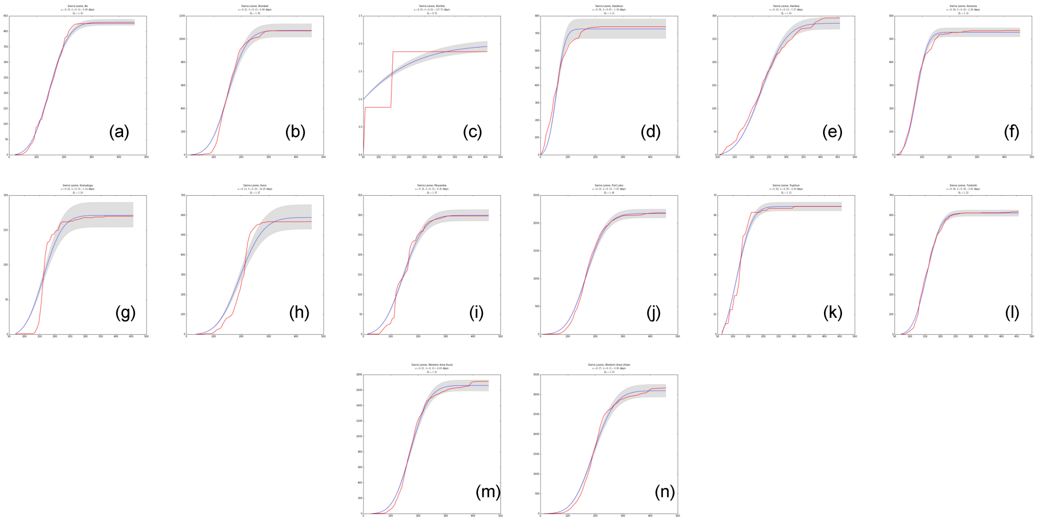

Figure S5. The best fit for the SDIR model for all first administrative level divisions in Sierra Leone. In order, these are: (a) Kailahun, (b) Kenema, (c) Kono Eastern, (d) Bombali, (e) Kambia, (f) Koinadugu, (g) Port Loko, (h) Tonkolili, (i) Bo, (j) Bonthe, (k) Moyamba, (l) Pujehun, (m) Western Rural, and (n) Western Urban.
